# Treatment of spinal cord injury, by restoration of neuronal networks using a combination of surgery and KCL-286, an orally available retinoic acid receptor β drug

**DOI:** 10.3389/fsurg.2026.1743444

**Published:** 2026-02-23

**Authors:** Thomas Carlstedt, Jonathan P. T. Corcoran

**Affiliations:** Neuroscience Drug Discovery Unit, Wolfson Sensory, Pain and Regeneration Centre, King’s College London, Guy’s Campus, London, United Kingdom

**Keywords:** avulsion brachial plexus injury, drug, nuclear receptor signalling, orally available drug, retinoic acid, spinal cord injury

## Abstract

The most complicated nerve injuries occur in the spinal nerves. Following traumatic injury at the nerve root attachment to the spinal cord (avulsion), there is degeneration of nerve fibres in the root and spinal cord. The result for the patient is paralysis with sensory loss and typical excruciating severe pain. An obvious fundamental surgical treatment for such injuries is to re-create continuity for the ruptured spinal nerve and the roots detached from the spinal cord. Reimplanting motor spinal roots leads to neuronal growth within the spinal cord and distally in the peripheral nerves, resulting in recovery of shoulder and proximal arm muscles. Sensory function cannot be restored surgically from dorsal root to spinal cord replantation due to the impediment of regrowing sensory fibres at the spinal cord glial scar*.* However, when a ganglionectomised dorsal root—in effect a peripheral nerve conduit—is implanted into the spinal cord sensory system, intramedullary (secondary) sensory neurons extend distally, resulting in recovery of some sensory function. Patients have profited from this surgery with better functional performance without movement synkinesis and reduced pain. Full functional restoration after a nerve injury cannot be achieved by means of surgery alone due to the impediment of regrowing sensory fibres at the spinal cord glial scar. Embryonic axogenesis was studied to identify pathways required for adult spinal cord injury repair. From this, a key regulator, the retinoic acid receptor β, was identified. This signalling cascade can be reactivated in the injured adult nervous system with the orally available drug KCL-286. This drug has been shown to be safe and tolerated in humans at doses predicted to be used in human spinal cord injuries to provide functional recovery. Therefore, the combination of surgical root implantation and KCL-286 represents a promising therapeutic strategy to improve the quality of life for patients with root avulsions and the broader population of patients with spinal cord injuries.

## Introduction

Even with the best and most up-to-date surgical treatment techniques, recovery after an injury to the nervous system is often far from normal. Some return of function can be noted after the repair of lesions in the peripheral nervous system, but hardly in the central nervous system (CNS). The compromising conditions include neuronal cell death, impaired nerve cell ability to regenerate, slow pace of neurite outgrowth, misdirection of growing axons, and curtailed growth in scar tissue. These conditions vary in the PNS and CNS and arise in different magnitudes but are common in the entire nervous system (1).

An intraspinal nerve injury is a lesion where the nerves linking the spinal cord to the muscles under voluntary control, or to various sensory receptors in the skin or subcutaneous tissue, have ruptured or been avulsed from the spinal cord. This is the most complicated and difficult nerve injury to repair and occurs most often in conjunction with nerve plexus trauma (1). The brachial plexus is little protected from traction forces because of the loose suspension of the shoulder girdle. Avulsion of at least one spinal nerve root of the brachial plexus occurs in approximately 70% of all brachial plexus lesions (2). Located within the bony pelvis, the lumbosacral plexus is more protected and spinal nerve root ruptures are more common than root avulsion injuries (3). These lesions are combinations of PNS and CNS injuries, particularly when one or several spinal nerve roots have ruptured or been torn or avulsed from their attachment to the spinal cord (2, 4). A root avulsion injury is a CNS lesion, and therefore not considered amenable to treatment as nerve fibres must regrow within the spinal cord (5). Surgical restoration of function and control of pain are formidable and frustrating tasks in patients suffering from such nerve injuries. The first description of intraspinal root repair is of clinical cases with conus tumours (6). Reattaching avulsed dorsal roots to the spinal cord was first described by Bonney and Jamieson (7). The first and, presently, only spinal cord surgical methods to restore regenerative function after spinal nerve root avulsion injury from the spinal cord were presented in 1995 (8, 9). This new and innovative surgery was developed following an extensive series of experimental laboratory procedures conducted in rats, cats, and the primate *Macaca fascicularis* (10).

### The spinal nerve injury

A severed ventral root—without an avulsion injury—is a peripheral nerve injury with good possibilities to regenerate (11). A severed dorsal root—without an avulsion injury—is considered a type of central nervous injury as sensory nerve fibre regrowth cannot occur across the root–spinal cord junction or the PNS–CNS transitional region. Because of this, it is considered the most common spinal cord injury (SCI) (12).

Root avulsions interrupt transverse segmental nerve fibres in the spinal cord, causing a “longitudinal spinal cord injury.” If left untreated, the affected spinal cord segments can deteriorate within approximately 1 month. There is the disintegration of neuronal networks and the death of motor (13), secondary sensory (14), and autonomic (15) neurons, leading to paralysis, loss of sensation, and excruciating, almost unbearable, severe intractable pain (16).

### Diagnosis

The diagnosis of a complete intraspinal nerve plexus avulsion injury by clinical and/or auxiliary means is difficult. Lesions can be situated simultaneously at multiple levels in both the spinal root and the peripheral nerve. There can be a mixture of motor and sensory root injuries with continuity severed in one or the other. Clinical assessment and neurophysiological testing can lead to erroneous diagnoses. Even the most sensitive MRI techniques provide false results in approximately 50% of cases. Computed tomography (CT) myelogram is still considered to be the gold standard for the preoperative evaluation of root avulsion (10).

Intraspinal endoscopy can provide more information regarding the remaining intradural stumps or full root avulsions (10). Direct inspection of the subdural space after laminectomy gives an accurate diagnosis of the extent of the root rupture or avulsion (17).

### Laboratory experimental surgery

In a long series of animal experiments, a direct, curative surgical technique for nerve root avulsions for clinical application in spinal cord surgeries was developed.

An original observation was made that spinal cord motoneurons, after ventral root avulsion and implantation, are reintegrated into local spinal cord reflex circuits and are able to regenerate and reinnervate muscles*.* Motoneurons have been observed by means of intracellular neurophysiology and electron microscopy to respond to injury by extending axons and dendrites (dendraxons) (18, 19).

Sensory function cannot be regained by implanting dorsal roots into the spinal cord. Dorsal root ganglia (DRG) neurons are unable to regrow axons into the CNS unless adjuvant molecules have been used (20). Like spinal cord motoneurons, spinal cord secondary sensory neurons can elongate new processes after injury. Provoked by an implanted PNS conduit, they can extend axons into a nerve graft after dorsal root ganglionectomy (10). Such recruited secondary sensory spinal cord neurons, many located in the lateral spinal nucleus (LSN), have connections for proprioception. MRI tractography demonstrated new neurite growth from the LSN into the periphery of importance for proprioception, as shown by electrophysiology (21). These findings prompted further experimental surgery and, eventually, human clinical application (10).

### Surgical management

A surgical approach in the lateral triangle of the neck and the cervical spine allows access to both the intra- and extraspinal parts of the brachial plexus [for surgical details, see (22)]. The intraspinal parts of the lumbosacral plexus and the cauda equina are exposed with the patient in the prone position [see (23)].

Medullary implantation of peripheral nerve grafts or reimplantation of roots into the spinal cord is performed through small slits in the pia mater in the ventro-lateral or dorso-lateral aspect of the spinal cord (17). The superficial position of the implanted nerve or root just below the pia mater is retained by means of tissue glue. During this procedure, spinal cord monitoring is performed to avoid any medullary dysfunction caused by the spinal cord surgery. If performed within 1 month after the injury, this surgery can promote the regrowth of new motor, sensory, or autonomic axons through spinal cord tissue (CNS regeneration). This regenerative effect is crucial for restoring nerve fibre trajectories and, hence, for the possible recovery of function and alleviation of pain (24). Reimplantation into the spinal cord, however, should not be considered in cases later than 1 month after injury (25).

### Functional results

Longitudinal assessments of restored activity were performed by means of functional MRI, transcranial magnetic stimulation, electromyography, and quantitative sensory testing of all sensory modalities, including sudomotor function. Recovery of motor function after spinal cord replantation occurs in a proximodistal direction and is strongest in the shoulder, upper arm muscles, and hip and thigh muscles after repair of intradural lumbar nerve roots (10). This postoperative functional gain in daily life refers to the arm being used as a prop for bilateral activities and the leg for standing and independent locomotion (10). The best recovery was noted in preadolescent children. These patients showed useful distal return of independent function in the forearm and hand (26). Functional outcomes after surgery can be misinterpreted and attributed to other conditions, such as plasticity, collateral sprouting, or anatomical variances, rather than from spinal cord regeneration, even when a complete brachial plexus avulsion injury had been verified at surgery. Prefixed or postfixed plexus anomalies are rare and would be unlikely to affect the outcome in a case where the entire brachial plexus is dislodged from the cervical spine and pulled underneath the clavicle. Experimental studies on collateral sprouting from adjacent intact nerves have failed to demonstrate convincing growth following experimental injuries (27).

To rule out sprouting in humans, recovery of function was assessed 3 years after spinal cord root implantation surgery by CT-guided root block of neighbouring non-avulsed roots. This study verified new axon growth through the spinal cord and into the root implants (8).

### Concomitant effects from injury and repair

**Figure 1:**
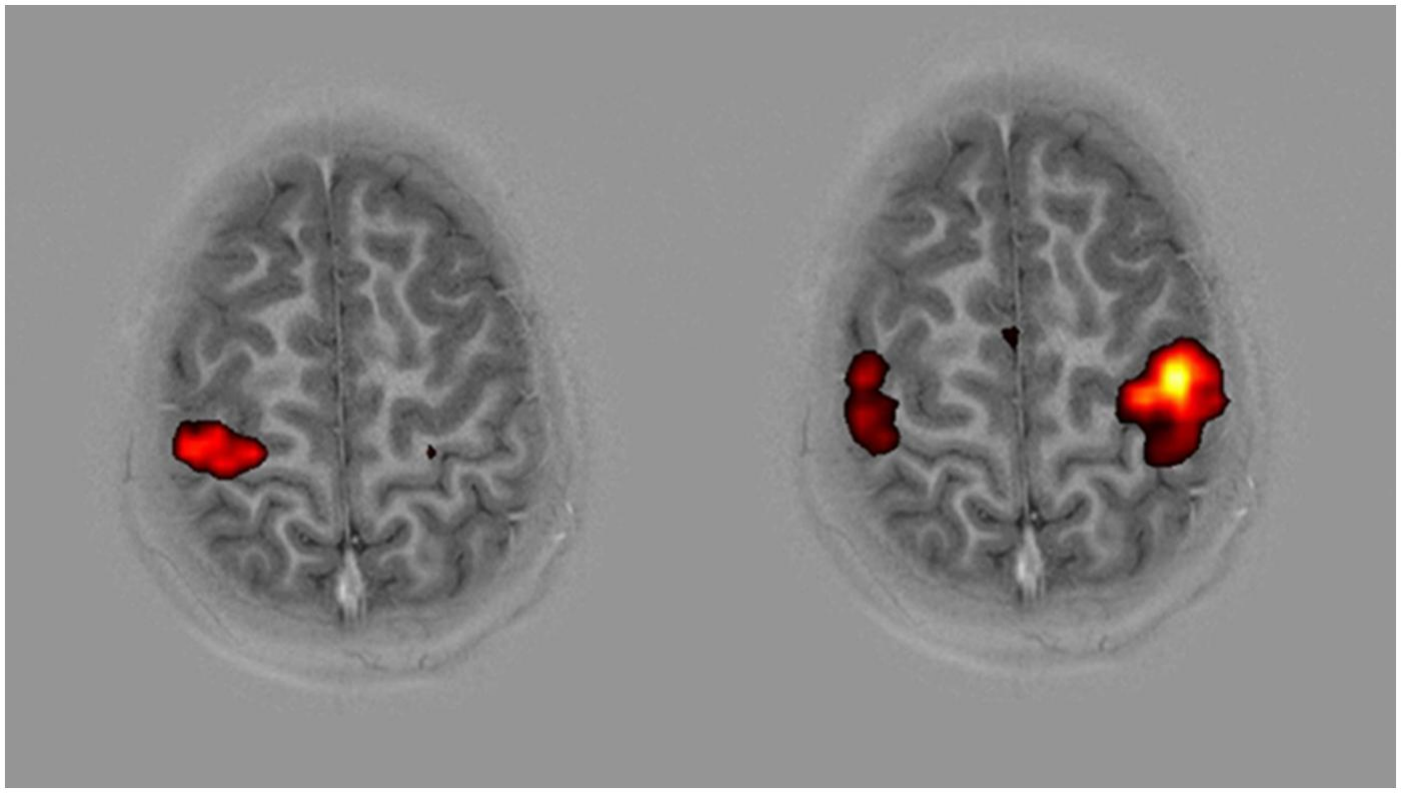
fMRI: axial image. Localisation of motor cortex (precentral gyrus) and sensory cortex (postcentral gyrus) determined by the omega shape of the central sulcus. Cortical activity from hand-squeezing a rubber ball. Normal hand (left picture). Activity only in the contralateral hemisphere. Affected hand (right picture). Sensory motor activity in both hemispheres (26).

The central nervous system can become structurally and functionally reorganised from a severe brachial plexus injury and spinal cord surgery. After ventral root to spinal cord replantation, functional MRI (fMRI) revealed expected motor cortex activity in the contralateral hemisphere of the operated limb. When the affected arm was used, activity was observed also on the ipsilateral side (Figure 1), which was not seen when the normal (intact) side was used (26). The ipsilateral cortex activity is most likely to be the result of cortical plasticity caused by post-injury diminished transcallosal inhibition (28), as has been noted in some CNS disorders (29). Only contralateral motor cortex plasticity has previously been observed on fMRI after peripheral nerve surgery (30). fMRI results also revealed that activity was observed in sensory cortex areas when the affected arm was used (9), despite the absence of sensory function. This probably occurs through pre-existing cortical sensory control in the associated sensory cortex areas (31) or from activation of some motoneurons located in the sensory cortex area. Spontaneous contractions of limb muscles in synchrony with respiration, i.e., “the breathing arm,” were noted in some operated cases after spinal cord repair of root avulsions. Such synkinesis was observed only during inspiration and verified by electromyography (EMG). The sources of this activity are the phrenic nerve motor neurons, which are situated at the C3–C5 spinal cord segments, in a discrete nucleus in the most medial part of the ventral horn, adjacent to the motoneurons supplying the shoulder and upper part of the arm. The implantation of a PNS conduit into the ventral part of the C-5 spinal cord segment could thus attract neurite growth from the phrenic nerve motor neurons within this segment to the arm instead of the diaphragm. This phenomenon demonstrates the intramedullary and spinal-cord-to-peripheral-nerve neurite growth produced by this surgery (32).

The quality of muscle function in many operated cases is disturbed by muscle co-contractions and synkinesis, probably due to absent sensory feedback and ineffective proprioception. In cases subjected to medullary connection of sensory neurons to the periphery, patients showed more controlled movements without the co-contractions and synkinesis only after the motor conduits had been repaired. Proprioception can be demonstrated by a bicep tendon reflex and electrophysiological demonstration of an H or Hoffman reflex (17). Functional MRI showed extended sensory motor cortical activity during active and passive arm movements in the primary somatosensory cortex S1 (9). The patient had perception of their movements but, in contrast to proprioception, there was no exteroception that could be demonstrated by objective sensory or cortical registration of contact evoked potential stimulation (9).

Dorsal root avulsion often generates spontaneous intractable deafferentation pain that parallels the generation of abnormal activity within the dorsal horn of the spinal cord. The magnitude of this pain is related to the number of avulsed roots (33). Pain provoked by light touch or allodynia experienced at the border zone of avulsed and non-avulsed dermatomes is probably due to reorganisation in the dorsal horn, for instance, inhibition of spinal afferent input, leading to spinal cord hyper-excitability (34). Reactive gliosis due to microglia and astrocyte activation (35) and metabolic changes, as revealed by proton magnetic resonance spectroscopy (36), in affected and neighbouring spinal cord segments could also cause the avulsion-induced pain. Relief of pain was frequently reported after timely repair of avulsed roots with reimplantation (33). Interestingly, alleviation of pain was also noted in conjunction with the return of motor function, probably due to the re-establishment of inhibitory cortical connections (26).

A majority of patients with dorsal root avulsions reported a referral of sensation that was perceived to be abnormal or at remote sites. Reorganisation of the somatosensory cortex by expansion of the adjacent sensory area into the deafferented cortex or in the thalamus may explain referred touch sensation from the face to the denervated arm (37). Sensation perceived in the deafferented arm when the trunk or the face is touched has been termed “wrong way of referral of sensation.” This type of sensation is considered to be related to plasticity in the cortex and is different from the “right way” of referred sensation, which can occur following peripheral nerve regeneration and is perceived at the origin of a lesioned nerve (33).

Surgery on spinal cord injuries sustained in conjunction with an intraspinal nerve injury has had promising results, demonstrating exceptional functional outcomes resulting from nerve fibre growth in the CNS (8). However, this recovery is limited and often disturbed by misdirected and erroneous connections caused by both the injury and surgery (33, 38). Restitution is particularly limited in the sensory system, occasionally leading to impeded motor functions, which has led to the current provisional method of implanting ganglionectomised dorsal roots. This outcome is, however, the best possible with surgery alone.

There is a need for non-surgical or adjuvant treatments to ameliorate outcomes after a spinal root avulsion in a longitudinal spinal cord lesion and in the treatment of a classical transverse spinal cord injury (39).

### The retinoic acid receptor β agonist drug KCL-286 is used to treat nerve injuries

#### Retinoic acid signalling during embryonic axogenesis

Retinoic acid (RA) signalling is essential for the development and maintenance of the CNS (40). One of the many examples of this is depriving adult hen quails of Vitamin A, which is ultimately converted into all trans RA (atRA). They can still lay fertile eggs and embryonic axogenesis can be followed, where it is shown that there are fewer neurons in the RA-deprived embryos that extend aberrant processes compared to normal embryos (41).

The RA signalling pathway consists of the retinoic acid receptors (RARs) and the retinoid X receptors (RXRs), of which there are three members, namely, *α*, *β*, and *γ*, and various subtypes (42). A heterodimer forms between RAR and RXR that binds to the retinoic acid response elements (RAREs) upstream of target genes, and when activated by the relatively non-selective ligand atRA, allows transcription to occur (43). Due to the number of pathways they regulate, they can be termed master regulators and the RARs and or RXRs that control axogenesis would be ideal candidates to target for nerve repair. The hierarchical function of the RARs/RXRs would also suggest that any mutations in them would be largely detrimental during embryogenesis and into adulthood. Consistent with this, gain-of-function mutations in human RARβ result in embryonic or neonatal mortality (44). In the limited cases where infants survive, they present with significant intellectual disability and a progressive decline in motor function (44).

This identification of a master regulator during embryonic axogenesis differs from genome-wide association studies (GWAS), which scan the genomes of many individuals to associate genetic variation with specific traits or diseases. Whereas GWAS often link single adult genetic variants to individual disease pathways, identifying a master regulator in embryonic axogenesis can reveal upstream control of multiple pathways. However, given the multifactorial nature of nerve injury, comprising both acute and chronic mechanisms, single targets identified in adults have not yielded clinical benefit (42). The embryonic regulators capable of coordinating multiple pathways are likely masked in humans because mutations in these regulators–whether loss- or gain-of-function–result in embryonic mortality.

To identify the RA signalling receptor for neurite outgrowth, mouse embryonic neurons were cultured in the presence of RAR and RXR agonists, which at that stage were tool compounds (cpds); only RARβ2 agonism was found to stimulate neurite outgrowth (45). Further confirmation of the importance of this receptor came from a study in cultured adult rodent CNSs, where it was found that RARβ2 was not expressed and no neurite outgrowth occurred in the presence of atRA. However, overexpression of RARβ2 by viral vectors induced some neurite outgrowth (46). This led to overexpression studies of RARβ2 in models of CNS injury where recovery occurred due to axonal outgrowth (47, 48). However, viral vectors have their own safety issues (49) and they require long-term follow-up (50), which, for a large unmet clinical need such as SCI, means that patient benefit may be some years away. In addition, with regard to gene therapy in the human adult injured spinal cord, it is unclear whether vectors can transduce a larger number of injured neurons in humans, as has been achieved in smaller rodent injuries.

Subsequent work showed that, unlike cultured CNS, RARβ was expressed in the injured adult PNS and CNS. This includes DRGs, motoneurons, and neuronal tracts in the spinal cord (39, 51). Using atRA or the RARβ tool cpd CD2019, regeneration of the nervous system has been demonstrated (52, 53), necessitating the need for development of a RARβ agonist drug to overcome issues with a gene therapeutic approach. One difference between the gene therapeutic approach taken and the use of an agonist is that in the former, a retinoid was not administered. It may be that the gene therapy approach allows the receptor to act like a transcriptional squelcher (54), sequestering available cofactors and, hence, the available RA, and turning off other pathways that may be regulated by the limited RA present that may be detrimental to axonal outgrowth. Due to the autoregulatory nature of RARβ2 (55), it can be deduced that once a certain level of transcripts is obtained by only using small molecules, a tipping point is reached that allows axonal regeneration to occur; thus, gene therapy and small molecule approaches are the same, as they both upregulate RARβ2.

### Drawbacks of current retinoid drugs and the development of KCL-286

The development of retinoids as therapeutics has been curtailed because most hit compounds have poor drug-like properties (56, 57), and the few lead compounds that progress to toxicity studies exhibit adverse events (AEs), the majority of which are related to liver toxicity due to their accumulation as fat-soluble molecules (42). Of the six retinoids that have been used in clinical settings, four are used to treat acne. These are the gel formulations adapalene and tretinoin and two orally available retinoids, isotretinoin and acitretin (42). As for the other two, tamibarotene is used to treat acute promyelocytic leukaemia and bexarotene, the only RXR agonist, to treat cutaneous T-cell lymphoma (42). What is also apparent about these drugs is that they are non-selective for RARβ, which, when tested in models of CNS injury, means they have no clear benefit (39).

The challenge then was to develop a selective RARβ agonist with good drug-like properties. From a hit to a lead compound, and following a lead-optimisation campaign, KCL-286 (C286) was developed. It fulfils key drug-development criteria, including oral availability, a clean profile in 28-day toxicity studies at the no-adverse-effect level (NOAEL) in rodent proof-of-concept (POC) nerve-injury studies, and good solubility and brain penetration (57). A phase I study in healthy male participants showed that the drug engaged its receptor and was well tolerated, with no drug-driven AEs at a 100-mg daily dose, equivalent to a 3-mg/kg oral dose in rodent POC studies on SCI (58).

### RARβ2 signalling is a master regulator of axogenesis

In rodent models of avulsion repair, in which severed sensory roots are reinserted into the spinal cord, treatment with KCL-286 administered orally every other day for 28 days (starting 1 day post-injury) successfully induced axons to form the correct connections within the dorsal horn (57). This functional reinnervation contrasts with human clinical outcomes, where sensory root implantation alone has failed to achieve similar restorative results (33, 38). The drug also resolved neuropathic pain in a spinal nerve ligation model (59). This efficacy stands in direct contrast to global RARα signalling, which has been identified as a mediator that increases neuropathic pain (60), thereby highlighting the necessity for specific RARβ agonists to treat nerve injuries. Furthermore, when using the same treatment regimen established for avulsion repair, C286 successfully prevented neuronal cell death and facilitated functional recovery in rodent models of spinal cord contusion, demonstrating its broader therapeutic applicability in various spinal cord injuries (39).

**Figure 2:**
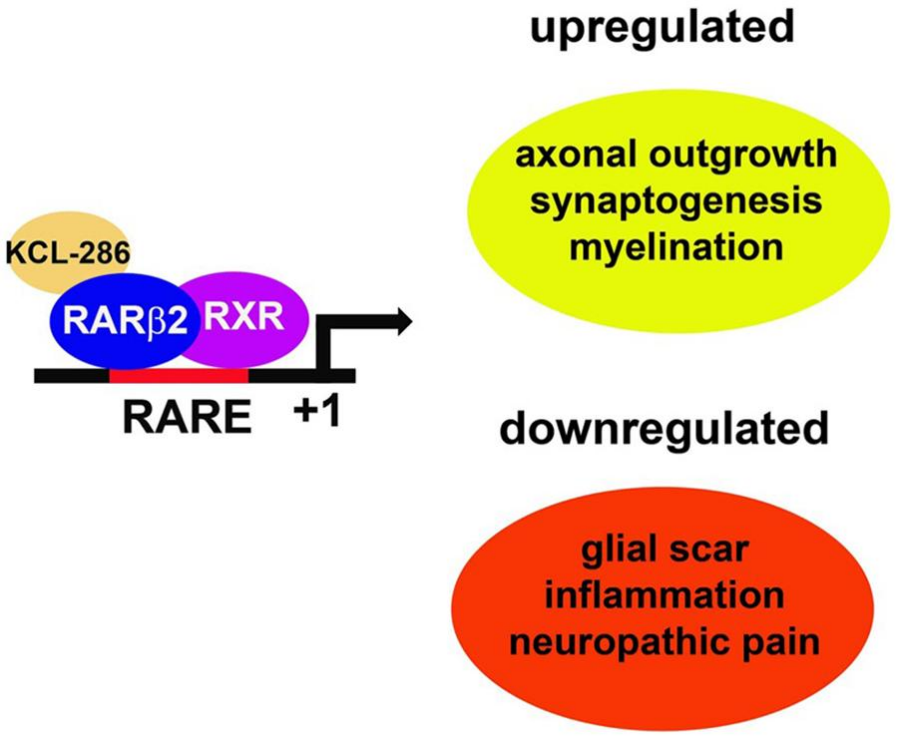
KCL-286 is a multifactorial drug for nerve repair. KCL-286 binds to a RARβ2/RXR heterodimer located at a retinoic acid response element (RARE). This results in the activation of the transcriptional pathways required for axonal regeneration (58).

As predicted, KCL-286 is multifactorial for nerve repair (Figure 2). It modulates the extracellular matrix, such as chondroitin sulphate proteoglycans, to create a permissive growth environment by removing the glial scar (39, 61). Moreover, it regulates myelination (61), reduces inflammation (39), and regulates the mitochondrial dynamics required to extend neurites (62). A pathway analysis of injured rodent spinal cords treated with KCL-286 revealed a multimodal regulatory effect across both neuronal and non-neuronal domains (39). These pathways have been identified in other studies of nerve repair, and include modulation of nicotinic receptors (63, 64), regulation of phagocytosis (65) and the orchestration of immune responses essential for axonal pathfinding (66, 67). A significant therapeutic advantage of this signalling is the initiation of an RARβ2-driven feedback loop, which sustains the transcriptional programmes necessary for axonal regeneration (20, 61).

Therefore, KCL-286 fulfils the criteria of a master regulator for nerve regeneration and may also be relevant to the treatment of neurodegenerative diseases, including Parkinson's disease, Alzheimer's disease, and motoneuron disease, given the correlation between RARβ expression and the affected neurons (39).

## Conclusion

We have discussed here the potential of a combination of surgery and KCL-286 to treat SCIs. Of note is that in human SCI surgery alone, there is an intrinsic capacity for functional nerve repair, and the drug boosts this potential in rodent models of SCI. The drug does not require extra surgical procedures, does not involve any known risks, is not a lifelong therapy, and is easy to administer. It is a drug that can target more than one source of regeneration failure with multifactorial functions and is potentially highly effective. The proposed Phase 2a clinical trial design entails surgery to be performed within 1 month after trauma. Ventral and dorsal roots are implanted superficially into the pia slits of the spinal cord. Since the drug promotes dorsal root axonal growth into the spinal cord across the glial scar, the dorsal roots are not ganglionectomised. KCL-286 is then given orally at a daily dose of 100 mg for 28 days. To monitor clinical efficacy, participants will undergo monthly functional assessments over 6 months to evaluate their sensory recovery, as the distance from the dorsal root implant to the dorsal horn is minimal in contrast to the motor recovery distance, which will still require a final follow-up at 18 months. In the eventual application and assessment of such a compound, the present functional outcomes and effects on central and peripheral nerve activity learned from the original application of spinal cord surgery in humans will no doubt serve as a baseline and be useful as a source of control data.
